# Meconium-Related Obstruction and Clinical Outcomes in Term and Preterm Infants

**DOI:** 10.1001/jamanetworkopen.2024.59557

**Published:** 2025-02-14

**Authors:** Jordan M. Rook, Nikhil Chervu, Kara L. Calkins, Peyman Benharash, Daniel A. DeUgarte

**Affiliations:** 1 Department of Surgery, UCLA David Geffen School of Medicine, Los Angeles, California; 2 Department of Health Policy and Management, UCLA Fielding School of Public Health, Los Angeles, California; 3 Cardiovascular Outcomes Research Laboratories, Department of Surgery, UCLA David Geffen School of Medicine, Los Angeles, California; 4 Division of Neonatology & Developmental Biology, Department of Pediatrics, Neonatal Research Center of the UCLA Children’s Discovery and Innovation Institute, UCLA David Geffen School of Medicine, Los Angeles, California; 5 Division of Pediatric Surgery, Department of Surgery, UCLA David Geffen School of Medicine, Los Angeles, California

## Abstract

**Importance:**

Textbooks attribute 80% of meconium-related small bowel obstructions to cystic fibrosis and 15% of colonic obstructions to Hirschsprung disease. It is unknown whether these estimates are accurate, particularly among preterm infants, whose immature bowel predisposes them to meconium-related obstruction (MRO).

**Objective:**

To estimate the incidence of MRO by type and to assess its association with clinical outcomes.

**Design, Setting, and Participants:**

This retrospective cohort study of live-born infants included in the National Inpatient Sample from January 1, 2016, to December 31, 2020, used survey weighting methods to estimate the national incidence of MRO by etiology. Data were analyzed from November 27, 2023, to November 12, 2024.

**Exposure:**

MRO.

**Main Outcomes and Measures:**

The primary outcome was diagnosis with MRO. Secondary outcomes included mortality, need for abdominal surgery, hospitalization duration, and cost. Multivariable regression models were developed to evaluate characteristics associated with MRO and to assess the association of MRO of prematurity with clinical outcomes after adjusting for demographic and clinical covariates.

**Results:**

Of 3 550 796 infants, 51.2% were male and 46.7% were privately insured. Overall, 9.1% (n = 322 499) were born preterm. Of 1844 (0.1%) infants treated for MRO, 41 (2.2%) had cystic fibrosis, 60 (3.3%) had Hirschsprung disease, and 1743 (94.5%) had neither predisposing condition. Preterm infants were at highest risk for MRO, with 4.7 MRO cases per 100 000 births associated with cystic fibrosis, 4.7 MRO cases per 100 000 births associated with Hirschsprung disease, and 187.3 MRO cases per 100 000 births associated with neither predisposing condition. Among infants with neither cystic fibrosis nor Hirschsprung disease, those with gestational ages from 28 weeks to 31 weeks 6 days were most likely to develop MRO compared with term infants (adjusted odds ratio, 6.08 [95% CI, 4.27-8.67]). Among preterm infants, having an MRO was associated with a 4.2 percentage point increase in the probability of abdominal surgery (95% CI, 3.1-5.4 percentage points), a 7.3-day increase in length of stay (95% CI, 5.8-8.8 days), and a $23 215 increase in hospitalization costs (95% CI, $17 739-$28 690) compared with infants who did not have an obstruction, with no change in mortality rate (0.1 percentage point change [95% CI, −0.6 to 0.8 percentage points]).

**Conclusions and Relevance:**

In this cohort study of over 3.5 million infants, MRO was most likely to occur among preterm infants without cystic fibrosis or Hirschsprung disease. These infants more frequently required surgery and had longer and more costly hospitalizations, indicating a need for dedicated prevention and treatment pathways for this understudied disease.

## Key Points

**Question:**

How often does meconium-related obstruction (MRO) occur without predisposing cystic fibrosis or Hirschsprung disease, and are these obstructions associated with clinical outcomes?

**Findings:**

In this cohort study of 3 550 796 live-born infants, 94.5% of MRO cases occurred in the absence of cystic fibrosis or Hirschsprung disease, with the highest incidence among preterm infants. Among preterm infants, MRO was associated with more frequent abdominal surgery and longer and more costly hospitalization compared with unaffected infants.

**Meaning:**

Findings from this cohort study suggest that MRO is most likely to occur in preterm infants without cystic fibrosis or Hirschsprung disease and that dedicated prevention, diagnostic, and treatment pathways are needed.

## Teaser

This cohort study uses a nationally representative dataset of hospitalizations in the US to investigate the incidence of meconium-related small bowel obstruction that occurs without predisposing cystic fibrosis or Hirschsprung disease and to assess the association of this obstruction with clinical outcomes among live-born infants.

## Introduction

Meconium-related obstruction (MRO) entails an intestinal blockage associated with thick inspissated meconium.^1,2^ These cases have historically been stratified by the level of obstruction, with meconium ileus describing small bowel obstructions and meconium plug syndrome describing colonic obstructions.^1,3^ Most textbooks attribute 80% of cases of meconium ileus to cystic fibrosis and 15% of cases of meconium plug syndrome to Hirschsprung disease.^4,5,6,7,8,9^ Thus, a diagnosis of MRO increases suspicion of underlying cystic fibrosis or Hirschsprung disease.^5,10^

Several case series have described MRO in the absence of cystic fibrosis or Hirschsprung disease.^2,4,11,12^ This type of obstruction, hereafter referred to as MRO unspecified, is more common in preterm infants and thought to be due to weak peristalsis and dysmotility.^2^ It can occur in both the small bowel and colon and is reported to require surgery in approximately one-third of cases.^2,11,12^ Beyond these estimates, little is known regarding the consequences of these obstructions for neonates and health care systems.

To our knowledge, no study has defined the modern epidemiology of MRO nor evaluated differences in disease incidence for preterm infants. It is unknown what proportion of infants treated for MRO may have underlying cystic fibrosis or Hirschsprung disease. Epidemiological data could inform clinical suspicion for these diseases and guide diagnostic workup. Given improving preterm infant survival, expanding limits of preterm viability, and decreasing national cystic fibrosis incidence, we hypothesized that most MROs occur in preterm infants with neither cystic fibrosis nor Hirschsprung disease.^13,14,15,16^ To test this hypothesis and better define this understudied disease, we used a nationally representative sample of infants to estimate the incidence of MRO; define rates of underlying prematurity, cystic fibrosis, and Hirschsprung disease; and evaluate changes in mortality, rates of surgery, and hospitalization costs and duration associated with MRO among preterm infants.

## Methods

### Data Source and Sample

This was a retrospective cohort study of the 2016 through 2020 National Inpatient Sample (NIS) Healthcare Cost and Utilization Project, maintained by the Agency for Healthcare Research and Quality.^17^ Each year the NIS includes approximately 7 million hospitalizations, comprising one-fifth of hospitalizations in the United States. After applying sampling weights, this dataset produces nationally representative estimates accounting for 98% of the US population.^18^ The use of the NIS for the study of pediatric diseases has been previously validated.^19,20^ The present study was deemed exempt from review by the University of California, Los Angeles, Institutional Review Board as a secondary data analysis using publicly available deidentified data. Thus, the need to obtain informed consent was also waived. The study was reported following the Strengthening the Reporting of Observational Studies in Epidemiology (STROBE) reporting guideline.

**Figure 1.**
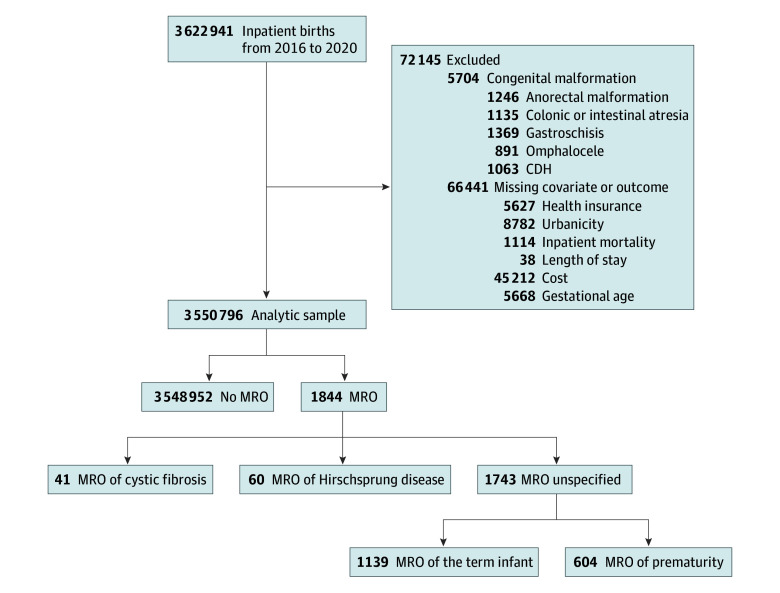
Flowchart Describing Cohort Construction and Meconium-Related Obstruction (MRO) by Type eTable 1 in Supplement 1 provides the *International Statistical Classification of Diseases, Tenth Revision*, diagnosis codes used for cohort construction. CDH indicates congenital diaphragmatic hernia.

We identified all neonatal hospitalizations using *International Statistical Classification of Diseases*, *Tenth Revision (ICD-10)*, diagnosis codes indicating an encounter for a live birth. We excluded infants with congenital defects that could result in neonatal intestinal obstruction. This included anorectal malformations, colonic or intestinal atresia, gastroschisis, omphalocele, and congenital diaphragmatic hernia (eTable 1 in Supplement 1). Last, we excluded records with any missing covariates or outcomes (Figure 1).

### Outcomes

The primary outcome was diagnosis with MRO. These cases were identified using *ICD-10* codes indicative of a bowel obstruction secondary to inspissated meconium. We used the term MRO instead of meconium ileus or meconium plug syndrome, as the level of obstruction cannot be determined from *ICD-10* codes. Cases were then stratified based on concomitant cystic fibrosis or Hirschsprung disease into the following 3 categories: (1) MRO of cystic fibrosis, (2) MRO of Hirschsprung disease, and (3) MRO unspecified (ie, occurring in the absence of cystic fibrosis or Hirschsprung disease). MRO unspecified was further stratified by prematurity into either MRO of the term infant or MRO of prematurity (eTable 1 in Supplement 1). We validated this identification strategy using institutional data and found that it was both highly sensitive (100%) and specific (94.8%) at identifying MRO among a cohort of neonates with gastrointestinal tract illness.^21^

To explore the relationship of MRO of prematurity with clinical outcomes, we assessed inpatient mortality, need for abdominal surgery, length of hospital stay, and hospitalization costs. Inpatient mortality was evaluated as a binary variable indicating death prior to discharge. Abdominal surgery was evaluated as a binary variable and included procedures likely to be performed for complications of MRO. These were identified using *ICD-10* procedure codes that included exploratory laparotomy, diagnostic laparoscopy, enterectomy, colectomy, and ostomy creation (eTable 2 in Supplement 1).^2,11,12,21^ Length of hospital stay was measured in days. Hospitalization costs were measured in US dollars and represented costs incurred by a hospital for services provided.^22^ All costs were inflation adjusted to 2020 values using the Consumer Price Index for All Urban Consumers.

### Covariates

We evaluated demographic variables, including race and ethnicity, biological sex, health insurance, urbanicity, and census region. Race and ethnicity were evaluated to explore the social factors associated with MRO, were assessed as reported in the NIS dataset as a categorical variable, and included Asian or Pacific Islander, Black, Hispanic, Native American, White, other, and unknown.^17^ The method for collecting race and ethnicity data is not specified by the NIS, as collection methods vary by hospital.^23^ We evaluated clinical characteristics including prematurity, birth weight, and small for gestational age (<10th percentile birth weight). Neonates were categorized as extremely preterm if estimated gestational age was less than 28 weeks, very preterm from 28 weeks through 31 weeks 6 days, moderately preterm from 32 weeks through 33 weeks 6 days, and late preterm from 34 weeks through 36 weeks 6 days.^24,25^ Neonates were categorized as extremely low birth weight if they were less than 1000 g, very low birth weight if 1000 to 1499 g, low birth weight if 1500 to 2499 g, and normal birth weight if at least 2500 g.^26^

For our analyses assessing mortality, morbidity, length of hospital stay, and costs of care, we assessed additional covariates associated with medical complexity, including concomitant respiratory distress syndrome, critical congenital heart disease, and grade 2 or greater intraventricular hemorrhage (eTable 1 in Supplement 1).^27^ Critical congenital heart disease included hypoplastic left heart syndrome, pulmonary atresia, tetralogy of Fallot, total anomalous pulmonary venous return, transposition of the great arteries, tricuspid atresia, and truncus arteriosus.^28^

### Statistical Analysis

Analyses were performed from November 27, 2023, to November 12, 2024, using Stata, version 17.0 (StataCorp LLC) and used a 2-sided α level of .05. We compared infants who developed MRO with those who did not using χ^2^ tests for categorical variables and analysis of variance for continuous variables. We used survey weights to produce nationally representative estimates of disease incidence for MRO by etiology. Estimates were further stratified by prematurity and birth weight. This process was repeated for the subset of infants with MRO who required surgical intervention. Incidence was reported in cases per 100 000 at-risk live births.

We used multivariable logistic regression analysis to assess characteristics associated with the diagnosis of MRO unspecified. Models assessed race and ethnicity, sex, degree of prematurity, birth weight, and small for gestational age. We then used multivariable logistic regression to assess differences in rates of mortality and surgery by the presence of MRO of prematurity. We used negative binomial regression to assess differences in length of hospital stay and a generalized linear model with a log link and gamma distribution to assess differences in cost. Models were adjusted for race and ethnicity, biological sex, health insurance, prematurity, birth weight, small for gestational age, comorbid illnesses, urbanicity, census region, and year. All regression outputs were converted to marginal estimates of effect. To assess data quality, we compared the incidence of prematurity, low birth weight, cystic fibrosis, MRO of cystic fibrosis, and Hirschsprung disease in our sample to previously published national estimates (eTable 3 in Supplement 1).^15,29,30^

We conducted several sensitivity analyses. First, to ensure that potential cases of MRO associated with cystic fibrosis or Hirschsprung disease were not inappropriately excluded, we assessed the co-occurrence of these diseases with all *ICD-10* codes indicative of bowel obstruction or ileus (eTable 4 in Supplement 1). Furthermore, to evaluate potential bias due to complete case analysis, we assessed the frequency of MRO among infants excluded for missing data (eTable 5 in Supplement 1).

## Results

### Sample Characteristics

Across the 5-year study period, we identified 3 550 796 neonates representing nearly 18 million live births. Among these, 188 828 (5.3%) were non-Hispanic Asian or Pacific Islander; 477 127 (13.4%) were non-Hispanic Black; 640 044 (18.0%) were Hispanic; 23 176 (0.7%) were non-Hispanic Native American; 1 649 217 (46.4%) were non-Hispanic White; 230 673 (6.5%) were characterized as other, which included patients who identified as other race or ethnicity or as not specified by the NIS classification; and 341 731 (9.6%) had unknown race and ethnicity. Overall, 1 816 310 (51.2%) were male and 1 734 486 (48.8%) were female. Infants most often had private insurance (1 658 332 [46.7%]). In total, 322 499 (9.1%) infants were born preterm, and 230 371 (6.5%) were born with low birth weight (Table 1).

**Table 1.** Patient Characteristics by Diagnosis of Meconium-Related Obstruction, 2016 to 2020

Characteristic	Patients, No. (%)			*P* value
	Total (N = 3 550 796)^a^	No MRO (n = 3 548 952)	MRO (n = 1844)	
Total No.		3 548 952	1844	
Cystic fibrosis	118 (<0.1)	77 (<0.1)	41 (2.2)	<.001
Hirschsprung disease	341 (<0.1)	281 (<0.1)	60 (3.3)	<.001
Race and ethnicity				
Asian or Pacific Islander	188 828 (5.3)	188 766 (5.3)	62 (3.4)	<.001
Black	477 127 (13.4)	476 612 (13.4)	515 (27.9)	
Hispanic	640 044 (18.0)	639 808 (18.0)	236 (12.8)	
Native American	23 176 (0.7)	≥23 166 (0.7)^b^	≤10 (<1.0)^c^	
White	1 649 217 (46.4)	1 648 467 (46.4)	≥746 (>40.4)	
Other^d^	230 673 (6.5)	230 556 (6.5)	117 (6.3)	
Unknown	341 731 (9.6)	341 573 (9.6)	158 (8.6)	
Sex				
Female	1 734 486 (48.8)	1 733 601 (48.8)	885 (48.0)	.46
Male	1 816 310 (51.2)	1 815 351 (51.2)	959 (52.0)	
Insurance				
Private	1 658 332 (46.7)	1 657 528 (46.7)	804 (43.6)	<.001
Medicare	10 045 (0.3)	≥10 035 (0.3)	≤10 (<1.0)	
Medicaid	1 616 694 (45.5)	1 615 754 (45.5)	≥937 (>50.0)	
Uninsured	169 585 (4.8)	169 530 (4.8)	55 (3.0)	
Other payer	96 140 (2.7)	96 102 (2.7)	38 (2.1)	
Prematurity				
Extremely (<28 wk)	19 855 (0.6)	19 768 (0.6)	87 (4.7)	<.001
Very (28-31wk 6 d)	31 499 (0.9)	31 356 (0.9)	143 (7.8)	
Moderately (32-33wk 6d)	40 931 (1.2)	40 852 (1.2)	79 (4.3)	
Late preterm (34w-36 wk 6d)	230 214 (6.5)	229 889 (6.5)	325 (17.6)	
Term (≥37 wk)	3 228 297 (90.9)	3 227 087 (90.9)	1210 (65.6)	
Birthweight				
Extremely low (<1000 g)	19 095 (0.5)	18 967 (0.5)	128 (6.9)	<.001
Very low (1000-1499 g)	24 178 (0.7)	24 083 (0.7)	95 (5.2)	
Low (1500-2499 g)	187 098 (5.3)	186 858 (5.3)	240 (13.0)	
Normal (≥2500 g)	3 320 425 (93.5)	3 319 044 (93.5)	1381 (74.9)	
Small for gestational age				
No	3 408 750 (96.0)	3 407 056 (96.0)	1694 (91.9)	<.001
Yes	142 046 (4.0)	141 896 (4.0)	150 (8.1)	
Respiratory distress syndrome				
No	3 353 877 (94.5)	3 352 481 (94.5)	1396 (75.7)	<.001
Yes	196 919 (5.5)	196 471 (5.5)	448 (24.3)	
Critical congenital heart disease				
No	3 547 202 (99.9)	3 545 361 (99.9)	≥1834 (>99.0)	.41
Yes	3594 (0.1)	≥3584 (0.1)	≤10 (<1.0)	
Intraventricular hemorrhage^e^				
No	3 546 190 (99.9)	3 544 367 (99.9)	1823 (98.9)	<.001
Yes	4606 (0.1)	4585 (0.1)	21 (1.1)	
Urbanicity				
Large metropolitan	2 029 853 (57.2)	2 028 807 (57.2)	1046 (56.7)	.18
Small or medium metropolitan	1 044 813 (29.4)	1 044 290 (29.4)	523 (28.4)	
Micropolitan or rural	476 130 (13.4)	475 855 (13.4)	275 (14.9)	
Census region				
New England	142 549 (4.0)	142 456 (4.0)	93 (5.0)	<.001
Mid-Atlantic	434 351 (12.2)	434 139 (12.2)	212 (11.5)	
East North Central	501 511 (14.1)	501 198 (14.1)	313 (17.0)	
West North Central	252 807 (7.1)	252 682 (7.1)	125 (6.8)	
South Atlantic	681 151 (19.2)	680 718 (19.2)	433 (23.5)	
East South Central	221 349 (6.2)	221 255 (6.2)	94 (5.1)	
West South Central	494 448 (13.9)	494 214 (13.9)	234 (12.7)	
Mountain West	256 148 (7.2)	256 036 (7.2)	112 (6.1)	
Pacific West	566 482 (16.0)	566 254 (16.0)	228 (12.4)	
Year				
2016	729 335 (20.5)	728 961 (20.5)	374 (20.3)	.65
2017	728 457 (20.5)	728 101 (20.5)	356 (19.3)	
2018	713 178 (20.1)	712 788 (20.1)	390 (21.1)	
2019	702 840 (19.8)	702 471 (19.8)	369 (20.0)	
2020	676 986 (19.1)	676 631 (19.1)	355 (19.3)	

Abbreviation: MRO, meconium-related obstruction.

^a^ Represents 17 753 971 births after survey weighting.

^b^ Data with a greater than or equal to symbol (≥) are presented as such to prevent calculation of adjacent cells with 10 or fewer observations per National Inpatient Sample (NIS) guidelines.

^c^ Cells with 10 or fewer observations are reported as ≤10 per NIS guidelines.

^d^ Other race and ethnicity includes patients who identified as other race or ethnicity or as not specified, by the NIS classification.

^e^ Includes grade 2 or greater intraventricular hemorrhage.

### MRO

Of 1844 (0.1%) patients diagnosed with MRO, 41 (2.2%) had concomitant cystic fibrosis, 60 (3.3%) had Hirschsprung disease, and 1743 (94.5%) had neither condition. Compared with unaffected infants, newborns who developed MRO were more often preterm (634 [34.4%] vs 321 865 [9.1%]; *P* < .001), low birth weight (463 [25.1%] vs 229 908 [6.5%]; *P* < .001), and small for gestational age (150 [8.1%] vs 141 896 [4.0%]; *P* < .001). They were also more often Black (515 [27.9%] vs 476 612 [13.4%]) and more frequently had comorbid diseases associated with prematurity (Table 1).

From 2016 through 2020, the incidence of MRO was 51.9 cases per 100 000 live births. Stratified by etiology, there were 1.2 cases of MRO of cystic fibrosis per 100 000 births, 1.7 cases of MRO of Hirschsprung disease per 100 000 live births, and 49.1 cases of MRO unspecified per 100 000 births. Among term infants, there were 0.8 cases of MRO of cystic fibrosis per 100 000 births, 1.4 cases of MRO of Hirschsprung disease per 100 000 births, and 35.3 cases of MRO unspecified per 100 000 births. Among preterm infants, there were 4.7 cases of MRO of cystic fibrosis per 100 000 births, 4.7 cases of MRO of Hirschsprung disease per 100 000 births, and 187.3 cases of MRO unspecified per 100 000 births. Regardless of prematurity or birth weight, MRO unspecified accounted for most MRO cases. The highest disease incidence for MRO unspecified was among infants born very preterm (438.1 cases per 100 000) and with extremely low birth weight (665.1 cases per 100 000). By comparison, there were only 39.1 cases of MRO unspecified per 100 000 infants with a normal birth weight.

**Figure 2.**
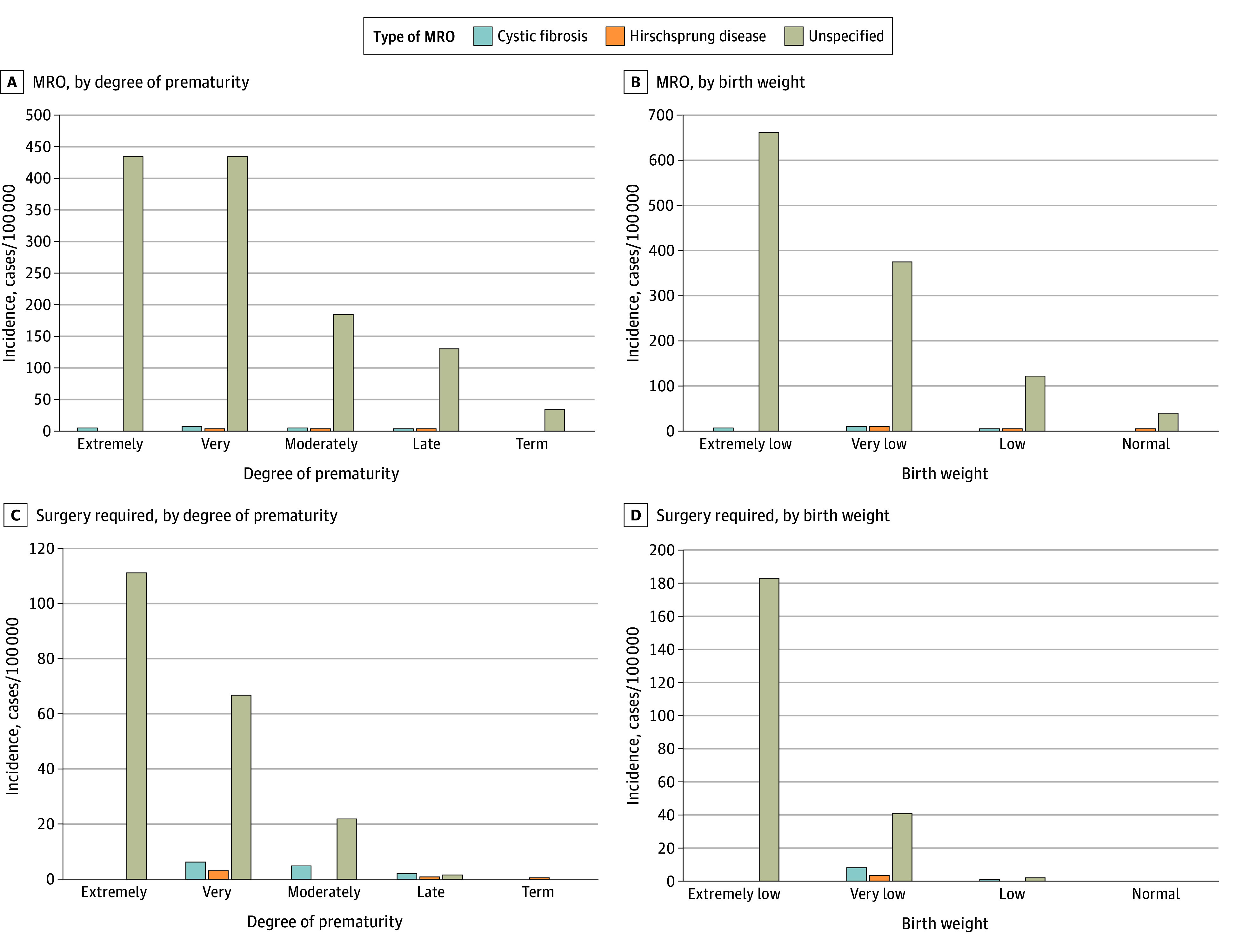
Incidence of Meconium-Related Obstruction (MRO) and MRO Requiring Surgery by MRO Type and Degree of Prematurity and Birth Weight eTable 6 in Supplement 1 provides calculated disease incidences. For degree of prematurity, extremely indicates less than 28 weeks; very, 28 weeks to 31 weeks 6 days; moderately, 32 weeks to 33 weeks 6 days; and late, 34 weeks to 36 weeks 6 days. For birth weight, extremely low indicates less than 1000 g; very low, 1000 to 1499 g; low, 1500 to 2499 g; and normal, at least 2500 g.

Of 98 patients who required surgical intervention, the etiology of MRO varied significantly by prematurity and birth weight (Figure 2; eTable 6 in Supplement 1). Among 41 term and late preterm infants with MRO who required surgery, 29 (70.7%) had concomitant cystic fibrosis or Hirschsprung disease. By comparison, of the 22 extremely preterm infants with MRO who required surgery, none had concomitant cystic fibrosis or Hirschsprung disease (*P* < .001). Among infants with MRO unspecified, frequency of surgery varied by prematurity. Of 1444 term and late preterm infants with MRO unspecified, 12 (0.8%) required surgery. By comparison, of 86 extremely preterm infants with MRO unspecified, 22 (25.6%) required surgery (*P* < .001).

### MRO Unspecified

On adjusted analyses, Black infants (adjusted odds ratio [AOR], 1.99 [95% CI, 1.77-2.24) were more likely than non-Hispanic White infants to develop MRO unspecified. All preterm infants were at higher risk for MRO unspecified than term infants, with gestational ages from 28 to 31 weeks 6 days conferring greatest risk (AOR, 6.08 [95% CI, 4.27-8.67]). Infants with extremely low birth weight (AOR, 5.21 [95% CI, 3.32-8.19]) and very low birth weight (AOR, 1.78 [95% CI, 1.21-2.60]) more often developed MRO than did infants with normal birth weight. Small for gestational age was independently associated with risk for obstruction (AOR, 1.66 [95% CI, 1.39-1.98]) (Table 2; eFigure in Supplement 1).

**Table 2.** Adjusted Association of Meconium-Related Obstruction Unspecified With Patient Characteristics^a^

Characteristic	AOR (95% CI)	*P* value
Race and ethnicity		
Asian or Pacific Islander	0.69 (0.52-0.91)	.009
Black	1.99 (1.77-2.24)	<.001
Hispanic	0.82 (0.71-0.96)	.02
Native American	0.38 (0.14-1.01)	.05
White	1 [Reference]	NA
Other^b^	1.11 (0.90-1.36)	.32
Unknown	1.02 (0.85-1.22)	.86
Sex		
Female	0.99 (0.91-1.09)	.82
Male	1 [Reference]	NA
Prematurity^c^		
Extremely	2.40 (1.44-3.98)	.001
Very	6.08 (4.27-8.67)	<.001
Moderately	4.41 (3.34-5.81)	<.001
Late preterm	3.57 (3.06-4.16)	<.001
Term	1 [Reference]	NA
Birth weight^d^		
Extremely low	5.21 (3.32-8.19)	<.001
Very low	1.78 (1.21-2.60)	.003
Low	0.95 (0.78-1.16)	.61
Normal	1 [Reference]	NA
Small for gestational age		
Yes	1.66 (1.39-1.98)	<.001
No	1 [Reference]	NA

Abbreviations: AOR, adjusted odds ratio; NA, not applicable.

^a^ Models adjust for race and ethnicity, sex, prematurity, birthweight, and small for gestational age.

^b^ Other race and ethnicity includes individuals who identified as other race or ethnicity or as not specified, by the National Inpatient Sample classification.

^c^ Extremely preterm, less than 28 weeks; very preterm, 28 weeks to 31 weeks 6 days; moderately preterm, 32 weeks to 33 weeks 6 days; late preterm, 34 weeks to 36 weeks 6 days.

^d^ Extremely low birth weight, less than 1000 g; very low birth weight, 1000 to 1499 g; low birth weight, 1500 to 2499 g; normal birth weight at least 2500 g.

### Clinical Outcomes of MRO of Prematurity

After adjustment for demographic and clinical characteristics, MRO of prematurity was associated with a 4.2 percentage point increase in the probability of abdominal surgery (95% CI, 3.1-5.4 percentage points; 1400.0% relative change), a 7.3-day increase in length of stay (95% CI, 5.8-8.8 days; 48.0% relative change), and a $23 215 increase in hospitalization costs (95% CI, $17 739-$28 690; 76.9% relative change) when compared with preterm infants without obstruction. MRO of prematurity was not associated with increased risk of mortality (0.1 percentage point change [95% CI, −0.6 to 0.8 percentage points]) (Table 3). By multiplying these average marginal effects by the weighted number of infants with MRO of prematurity from 2016 through 2020 (n = 3020), it is estimated that these cases accounted for 22 046 excess hospital days and $70 109 300 in excess costs during the 5-year study period.

**Table 3.** Adjusted Association of MRO of Prematurity With Abdominal Surgery, Mortality, Length of Hospital Stay, and Costs of Care for Preterm Infants^a^

Outcome	Adjusted outcomes by absence vs presence of MRO of prematurity		Adjusted change due to MRO of prematurity (95% CI)	Relative change, %^b^	*P* value
	No MRO of prematurity (95% CI)	MRO of prematurity (95% CI)			
Probability of surgery, %	0.3 (0.3-0.3)	4.5 (3.4-5.7)	4.2 (3.1 to 5.4)	1400	<.001
Mortality, %	2.3 (2.2-2.3)	2.3 (1.6-3.0)	0.1 (−0.6 to 0.8)	4.4	.82
Length of stay, d	15.2 (15.1-15.3)	22.5 (21.0-24.0)	7.3 (5.8 to 8.8)	48.0	<.001
Cost, $	30 180 (29 497-30 864)	53 395 (47 835-58 956)	23 215 (17 739 to 28 690)	76.9	<.001

Abbreviation: MRO, meconium-related obstruction.

^a^ MRO of prematurity is defined as an MRO occurring in a preterm neonate without cystic fibrosis or Hirschsprung disease. Models adjust for race and ethnicity, sex, insurance, prematurity, birth weight, small for gestational age, respiratory distress syndrome, critical congenital heart disease, grade 2 or greater intraventricular hemorrhage, urbanicity, census region, and year. All analyses account for survey weighting.

^b^ Relative change calculated by dividing the adjusted change due to MRO of prematurity by the estimate for individuals without MRO of prematurity (eg, calculation for length of stay, 7.3 / 15.2 = 0.48).

### Data Quality

For the entire sample, the incidences of prematurity (9082 cases per 100 000 births) and low birth weight (6488 cases per 100 000 births) were slightly lower than historical averages (10 230 cases per 100 000 births and 8310 cases per 100 000 births, respectively).^29^ While the incidences of cystic fibrosis and Hirschsprung disease were lower than historical incidences for the total sample, among preterm infants the calculated incidences were largely consistent with historical estimates (eTable 3 in Supplement 1).^15,30^ Sensitivity analyses assessing all *ICD-10* diagnosis codes for bowel obstruction identified no additional cases of MRO of cystic fibrosis and 27 cases of possible MRO of Hirschsprung disease (eTable 4 in Supplement 1). Among infants excluded for missing data, there was a slightly higher frequency of MRO in term infants (0.05% vs 0.03%) (*P* = .01; eTable 5 in Supplement 1).

## Discussion

This cohort study is the first, to our knowledge, to use nationally representative data to describe the epidemiology of MRO. We found that 94.5% of these obstructions occurred in the absence of cystic fibrosis or Hirschsprung disease. These MRO unspecified cases were more common among preterm infants and were associated with higher rates of abdominal surgery, longer hospitalizations, and greater costs of care. These findings indicate a need for dedicated investigation into diagnostic, prevention, and treatment pathways for this condition. Additional investigation is required to identify which preterm infants are at highest risk for this condition and to characterize current treatment strategies.

From January 1, 2016, to December 31, 2020, we identified 51.9 MRO cases per 100 000 live births. The overwhelming majority of these obstructions were MRO unspecified (ie, occurring in the absence of cystic fibrosis or Hirschsprung disease). The incidence of these obstructions was markedly higher among infants who were preterm or had low birth weight. For infants born with an extremely low birth weight, the incidence of MRO unspecified was 665.1 cases per 100 000 infants, a rate 17 times as high as infants born with normal birth weight. These findings are consistent with several small case series.^11,12^ MRO unspecified included a broad spectrum of disease severity that increased with prematurity. While less than 1% of term and late preterm infants with this condition required surgery, over a quarter of extremely preterm infants ultimately required an operation.

Regarding the pathophysiology of MRO unspecified, others^31^ have proposed poor intestinal motility and increased meconium viscosity as potential mechanisms by which obstructions develop. This is particularly relevant for preterm infants for whom intestinal immaturity results in these physiologic changes.^31,32^ We also found that small for gestational age status conferred additional risk for MRO unspecified regardless of prematurity or birth weight. Small for gestational age has previously been noted to be an independent risk factor for necrotizing enterocolitis, a disease also related to intestinal immaturity.^33^

Black infants were more likely to develop MRO even after adjusting for other clinical characteristics. This disparity is consistent with other disparities affecting Black women and their children, including higher maternal and infant mortality, rates of preterm birth, and small for gestational age.^34,35,36^ We hypothesize that social drivers of health and racism likely contribute to this difference in risk for MRO.^37,38^ These social drivers of health and racism include increased barriers to high-quality health insurance, lack of access to comprehensive prenatal care, chronic toxic stress, and implicit bias in health care.^39,40,41,42^ The identification and mitigation of these factors that result in disparate rates of MRO should be a target for health equity–focused research and quality improvement in our field.^39^

These broad epidemiological findings aid clinicians and researchers in identifying infants at high-risk for MRO such that prophylactic therapies can be developed. A recent meta-analysis reported prophylactic glycerin suppositories in preterm neonates to be associated with decreased time to meconium passage.^43^ Additionally, a recent single-center randomized clinical trial demonstrated reduced time to meconium evacuation and full enteral feeds among preterm neonates who received breast milk enemas.^44^ Ultimately, multi-institutional prospective trials are needed to develop a risk-based algorithm to identify and prophylactically treat preterm infants.

Among infants with MRO, cystic fibrosis and Hirschsprung disease were both rare, accounting for 2.2% and 3.3% of total obstructions, respectively. This overall frequency is much lower than prior studies, which report around 80% of small bowel obstructions to be secondary to cystic fibrosis^4,5^ and 13% to 15% of colonic obstructions to be secondary to Hirschsprung disease.^8,9^ This difference in findings may be in part due to our use of *ICD-10* codes that capture a broad spectrum of MROs, potentially reducing the relative frequency of clinically significant cases due to cystic fibrosis or Hirschsprung disease. This supposition is likely true for term and late preterm infants, among whom over half of patients requiring surgery had either cystic fibrosis or Hirschsprung disease. By comparison, among extremely preterm infants, no case of MRO requiring surgery was associated with these predisposing conditions. Among increasingly preterm infants, it may be that obstructions due to cystic fibrosis or Hirschsprung disease are simply overshadowed by the greater relative frequency of MRO unspecified in this population. Ultimately, future studies should evaluate diagnostic pathways for infants with MRO with the goal of reducing unnecessary testing and follow-up, particularly invasive procedures, such as suction rectal biopsy.^6,7,9^

This study also identified significant clinical implications for children treated for MRO of prematurity. These infants more frequently required surgery, had longer hospitalizations, and accumulated greater costs of care. Based on the findings of this study, from 2016 through 2020, MRO of prematurity accounted for over 22 000 excess hospital days and 70 million dollars in excess health care costs. These calculations do not account for the downstream effects of prolonged neonatal intensive care unit stays and neonatal surgery, both of which are deleterious to neurodevelopment.^45,46^

### Limitations

This study has several limitations. First, given that the NIS dataset samples only 20% of inpatient encounters, national estimates of incidence may be biased by sampling error.^17^ While this risk is minimized by a systematic sampling design, which reduces sampling bias by about half, potential for bias remains.^47^ Second, the use of *ICD-10* codes for cohort construction introduces potential measurement bias. This potential is particularly true for the study of cystic fibrosis and Hirschsprung disease given that these diagnoses are often confirmed after an infant’s newborn hospitalization. We sought to address this potential by testing various *ICD-10* code combinations and comparing the incidence of diseases derived from our sample with historical estimates. While comparative incidences were consistent among preterm infants, for the total sample, the incidences of cystic fibrosis and Hirschsprung disease were lower than expected. This finding may be attributable to pending workups and newborn screening at the time of hospital discharge. This limitation is less likely to affect preterm infants and infants with clinically significant MRO, as they require longer hospitalizations. These longer hospital stays may explain the better data quality identified among the preterm sample. Furthermore, diagnosis codes are likely to underestimate several covariates, including low birth weight and small for gestational age. Third, because the *ICD-10* diagnosis codes for MRO captures a spectrum of disease, including meconium ileus and meconium plug syndrome, it is plausible that this strategy overestimates the incidence of clinically significant disease. We conducted stratified analyses by need for surgery to better assess the most severe cases of MRO. Future studies should differentiate MRO by the level of obstruction to aid in the development of distinct diagnostic and treatment pathways.

## Conclusions

In this cohort study of over 3.5 million infants, we found that nearly 95% of MRO cases occurred in the absence of cystic fibrosis or Hirschsprung disease. Preterm infants and infants with low birth weight were at highest risk for this condition, with MRO of prematurity associated with an increased risk for abdominal surgery, longer hospitalizations, and greater costs of care. These findings call attention to this understudied disease. Future research should (1) identify high-risk infants, (2) evaluate prophylactic therapies, and (3) develop treatment pathways for infants with MRO. Through these efforts, we can reduce morbidity for preterm neonates as well as lessen strain on the pediatric health care system.

## Notes

Supplement 1. **eTable 1.** ICD-10 Diagnosis Codes Used for Covariate and Outcome Identification **eTable 2.** ICD-10 Procedure Codes Used to Identify Receipt of Abdominal Surgery **eTable 3.** Data Quality – Comparing Derived Disease Incidences to Historical Estimates **eTable 4.** Candidate ICD-10 Codes for Meconium-Related Obstruction and Frequency of Co-Occurrence with Cystic Fibrosis and Hirschsprung Disease **eTable 5.** Frequency of Meconium-Related Obstruction by Type Among Analytic Sample Versus Infants Excluded for Missing Data **eTable 6.** Incidence of Meconium Related Obstructions by Prematurity, Birth Weight, and Need for Surgery **eFigure.** Incidence of Meconium-Related Obstruction Unspecified by Small for Gestational Age Status and Degree of Prematurity and Birth Weight

Supplement 2.
Data Sharing Statement
