# Supplementary material for: Meconium-Related Obstruction and Clinical Outcomes in Term and Preterm Infants
Source: JAMA Netw Open. 2025 Feb 14;8(2):e2459557. doi: 10.1001/jamanetworkopen.2024.59557 (PMC11829230; doi:10.1001/jamanetworkopen.2024.59557)
Supplement: Supplement 1. — eTable 1. ICD-10 Diagnosis Codes Used for Covariate and Outcome Identification eTable 2. ICD-10 Procedure Codes Used to Identify Receipt of Abdominal Surgery eTable 3. Data Quality – Comparing Derived Disease Incidences to Historical Estimates eTable 4. Candidate ICD-10 Codes for Meconium-Related Obstruction and Frequency of Co-Occurrence with Cystic Fibrosis and Hirschsprung Disease eTable 5. Frequency of Meconium-Related Obstruction by Type Among Analytic Sample Versus Infants Excluded for Missing Data eTable 6. Incidence of Meconium Related Obstructions by Prematurity, Birth Weight, and Need for Surgery eFigure. Incidence of Meconium-Related Obstruction Unspecified by Small for Gestational Age Status and Degree of Prematurity and Birth Weight [file jamanetwopen-e2459557-s001.pdf]

Supplementary Online Content

Rook JM, Chervu N, Calkins KL, Benharash P, DeUgarte DA. Meconium-related obstruction and clinical outcomes in term and preterm infants. *JAMA Netw Open*. 2025;8(2):e2459557. doi:10.1001/jamanetworkopen.2024.59557

eTable 1. ICD-10 Diagnosis Codes Used for Covariate and Outcome Identification

eTable 2. ICD-10 Procedure Codes Used to Identify Receipt of Abdominal Surgery

eTable 3. Data Quality – Comparing Derived Disease Incidences to Historical Estimates

eTable 4. Candidate ICD-10 Codes for Meconium-Related Obstruction and Frequency of Co-Occurrence with Cystic Fibrosis and Hirschsprung Disease

eTable 5. Frequency of Meconium-Related Obstruction by Type Among Analytic Sample Versus Infants Excluded for Missing Data

eTable 6. Incidence of Meconium Related Obstruction by Prematurity, Birth Weight, and Need for Surgery

eFigure. Incidence of Meconium-Related Obstruction Unspecified by Small for Gestational Age Status and Degree of Prematurity and Birth Weight

This supplementary material has been provided by the authors to give readers additional information about their work.

© 2025 Rook JM et al. *JAMA Network Open*.

eTable 1. ICD-10 Diagnosis Codes Used for Covariate and Outcome Identification

**Cohort Construction**	ICD-10 Diagnosis Code	Description
Prematurity (<37 weeks EGA)		
36 weeks	P07.39	EGA 36 completed weeks
35 weeks	P07.38	EGA 35 completed weeks
34 weeks	P07.37	EGA 34 completed weeks
33 weeks	P07.36	EGA 33 completed weeks
32 weeks	P07.35	EGA 32 completed weeks
31 weeks	P07.34	EGA 31 completed weeks
30 weeks	P07.33	EGA 30 completed weeks
29 weeks	P07.32	EGA 29 completed weeks
28 weeks	P07.31	EGA 28 completed weeks
27 weeks	P07.26	EGA 27 completed weeks
26 weeks	P07.25	EGA 26 completed weeks
25 weeks	P07.24	EGA 25 completed weeks
24 weeks	P07.23	EGA 24 completed weeks
23 weeks	P07.22	EGA 23 completed weeks
< 23 weeks	P07.21	EGA less than 23 completed weeks
**Exclusion Criteria**		
Congenital Diaphragmatic Hernia	Q79.0	Congenital diaphragmatic hernia
	Q79.1	Other congenital malformations of the diaphragm
Omphalocele	Q79.2	Exomphalos
Gastroschisis	Q79.3	Gastroschisis
Duodenal/Jejunal/Ileal Atresia	Q41*	Congenital absence of small intestine
Colonic Atresia	Q42.8	Congenital absence, atresia, or stenosis of colon
	Q42.9	Congenital absence, atresia, or stenosis of colon, unspecified
Anorectal Malformation	Q42.0	Congenital absence, atresia, and stenosis of rectum with fistula
	Q42.1	Congenital absence, atresia, and stenosis of rectum without fistula
	Q42.2	Congenital absence, atresia, and stenosis of anus with fistula
	Q42.3	Congenital absence, atresia, and stenosis of anus without fistula
	Q43.6	Congenital fistula of rectum and anus
	Q43.7	Persistent cloaca

© 2025 Rook JM et al. *JAMA Network Open*.

**Meconium-Related Obstruction**		
Hirschsprung Disease	Q43.1	Hirschsprung disease
Cystic Fibrosis	E84*	Cystic fibrosis
MRO	P76.0	Meconium plug syndrome
MRO of Hirschsprung disease	P76.0 + Q43.1	Meconium plug syndrome with Hirschsprung disease
MRO of cystic fibrosis	E84.11	Meconium ileus in cystic fibrosis
	E84.19	Other intestinal manifestations of cystic fibrosis
	P76.0 + E84*	Meconium plug syndrome with cystic fibrosis
MRO of prematurity	P76.0 + P07.2 or P07.3 w/o E84.11 or Q43.1	Meconium plug syndrome with prematurity and without cystic fibrosis or Hirschsprung disease
MRO of the term infant	P76.0 w/o P07.2 or P07.3 w/o E84.11 or Q43.1	Meconium plug syndrome without prematurity, cystic fibrosis, or Hirschsprung disease
**Covariates**		
Low Birthweight		
2,000-2,499 grams	P07.14	Weight 2,000-2,499 grams
1,750-1,999 grams	P07.15	Weight 1,750-1,999 grams
1,500-1,749 grams	P07.16	Weight 1,500-1,749 grams
1,250-1,499 grams	P07.17	Weight 1,250-1,499 grams
1,000-1,249 grams	P07.18	Weight 1,000-1,249 grams
750-999 grams	P07.03	Weight 750-999 grams
500-749 grams	P07.02	Weight 500-749 grams
<500 grams	P07.01	Weight < 500 grams
Respiratory Distress Syndrome	P22.0	Respiratory distress syndrome of newborn
	P22.8	Other respiratory distress of newborn
	P22.9	Respiratory distress of newborn, unspecified
	P27.1	Bronchopulmonary dysplasia
	P27.8	Other chronic respiratory diseases originating in the perinatal period
	P27.9	Unspecified chronic respiratory disease originating in the perinatal period

© 2025 Rook JM et al. *JAMA Network Open*.

	P28.5	Respiratory failure of newborn
Intraventricular Hemorrhage	P52.1	IVH grade II
	P52.21	IVH grade III
	P52.22	IVH grade IV
Critical Congenital Heart Disease	Q20.0	Common arterial trunk
	Q22.4	Congenital tricuspid stenosis
	Q20.3	Discordant ventriculoarterial connection
	Q23.4	Hypoplastic left heart syndrome
	Q22.0	Pulmonary valve atresia
	Q21.3	Tetralogy of Fallot
	Q26.2	Total anomalous pulmonary venous connection
Small for Gestational Age	P05.0*	Light for gestational age
	P05.1*	Small for gestational age
	P05.9	Newborn affected by slow intrauterine growth

Abbreviations: MRO – meconium-related obstruction; EGA – estimated gestational age; IVH – intraventricular hemorrhage; ICD-10 – International Classification of Diseases, 10th revision. * - Indicates all codes starting with this prefix.

© 2025 Rook JM et al. *JAMA Network Open*.

eTable 2. ICD-10 Procedure Codes Used to Identify Receipt of Abdominal Surgery

**Abdominal Surgery**	ICD-10 Diagnosis Code	Procedure/Procedure Family
Exploratory laparotomy or diagnostic laparoscopy	0DJ* without *XZZ	Inspection of peritoneal cavity
	0WJP0ZZ	Inspection of GI tract, open
	0WJP4ZZ	Inspection of GI tract, percutaneous endoscopic
Small bowel resection	0DT8*	Resection of small intestine
	0DTA*	Resection of jejunum
	0DTB*	Resection of ileum
	0DTC*	Resection of ileocecal valve
	0DB8*	Excision of small intestine
	0DBA*	Excision of jejunum
	0DBB*	Excision of ileum
	0DBC*	Excision of ileocecal valve
Small bowel primary repair	ODQ8*	Repair of small intestine
	0DQA*	Repair of jejunum
	0DQB*	Repair of ileum
	0DQC*	Repair of ileocecal valve
Colectomy	0DTE*	Resection of colon
	0DTF*	Resection of right colon
	0DTG*	Resection of left colon
	0DTH*	Resection of cecum
	0DTK*	Resection of ascending colon
	0DTL*	Resection of transverse colon
	0DTM*	Resection of descending colon
	0DTN*	resection of sigmoid colon
	0DBE*	Excision of colon
	0DBF*	Excision of right colon
	0DBG*	Excision of left colon
	0DBH*	Excision of cecum
	0DBK*	Excision of ascending colon
	0DBL*	Excision of transverse colon
	0DBM*	Excision of descending colon
	0DBN*	Excision of sigmoid colon
Colon Primary Repair	0DQE*	Repair of colon
	0DQF*	Repair of right colon
	0DQG*	Repair of left colon
	0DQH*	Repair of cecum

© 2025 Rook JM et al. *JAMA Network Open*.

	0DQK*	Repair of ascending colon
	0DQL*	Repair of transverse colon
	0DQM*	Repair of descending colon
	0DQN*	Repair of sigmoid colon
Open or Laparoscopic Peritoneal Drain Placement	0D9W00Z	Drainage of peritoneum with drainage device, open approach
	0D9W0ZX	Drainage of peritoneum, open approach, diagnostic
	0D9W0ZZ	Drainage of peritoneum, open approach
	0D9W4ZX	Drainage of peritoneum, percutaneous endoscopic approach, diagnostic
	0D9W4ZZ	Drainage of peritoneum, percutaneous endoscopic approach
Jejunostomy or Ileostomy Creation	0D18***4	Bypass small intestine to cutaneous with autologous tissue substitute, open approach
	0D1A***4	Bypass jejunum to cutaneous with autologous tissue substitute, open approach
	0D1B***4	Bypass ileum to cutaneous with autologous tissue substitute, open approach
Colostomy	0D1E***4	Bypass colon to cutaneous with autologous tissue substitute, open approach
	0D1H***4	Bypass cecum to cutaneous with autologous tissue substitute, open approach
	0D1K***4	Bypass ascending colon to cutaneous with autologous tissue substitute, open approach
	0D1L***4	Bypass transverse colon to cutaneous with autologous tissue substitute, open approach
	0D1M***4	Bypass descending colon to cutaneous with autologous tissue substitute, open approach
	0D1N***4	Bypass sigmoid colon to cutaneous with autologous tissue substitute, open approach
Drain placement	0D9W00Z	Drainage of peritoneum, open with drainage device
	0D9W0ZZ	Drainage of peritoneum, open diagnostic
	0D9W0ZX	Drainage of peritoneum, open
	0D9W4ZX	Drainage of peritoneum, percutaneous endoscopic diagnostic
	0D9W4ZZ	Drainage of peritoneum percutaneous endoscopic
	0W9G00Z	Drainage of peritoneal cavity, open with drainage device
	0W9G0ZX	Drainage of peritoneal cavity, open diagnostic
	0W9G0ZZ	Drainage of peritoneal cavity, open
	0W9G4ZX	Drainage of peritoneal cavity, percutaneous endoscopic diagnostic
	0W9G4ZZ	Drainage of peritoneal cavity, percutaneous endoscopic

Abbreviations ICD-10 – International Classification of Diseases, 10th revision. * - Indicates all codes starting with this prefix.

© 2025 Rook JM et al. *JAMA Network Open*.

eTable 3. Data Quality – Comparing Derived Disease Incidences to Historical Estimates

	Annual Cases (2016-2020)	Database Incidence (Cases/100,000)	Historical Incidence (Cases/100,000)
Total Births (n)	3,550,796		
Preterm Birth	322,499	9,082	10,230 [a]
Low Birth weight	230,371	6,488	8,310 [a]
Cystic Fibrosis	118	3.3	19.6 [b]
MRO of Cystic Fibrosis	41	1.2	4.0 [b]
Hirschsprung Disease	341	9.6	22.1 [c]
MRO of Hirschsprung Disease [d]	60	1.7	N/A [d]
Total Preterm Births (n)	322,499		
Cystic Fibrosis	50	15.5	19.6 [b]
MRO of Cystic Fibrosis	15	4.7	4.0 [b]
Hirschsprung Disease	100	31.0	22.1 [c]
MRO of Hirschsprung Disease [d]	15	4.7	N/A [d]

[a] Martin J, Hamilton B, Osterman M, Driscoll A. Births: Final Data for 2019. *Natl Vital Stat Rep*. 2021;70(2):1-51. [b] Stephenson AL, Swaleh S, Sykes J, et al. Contemporary cystic fibrosis incidence rates in Canada and the United States. *J Cyst Fibros*. 2023;22(3):443-449. doi:10.1016/j.jcf.2022.10.008. [c] Anderson JE, Vanover MA, Saadai P, Stark RA, Stephenson JT, Hirose S. Epidemiology of Hirschsprung disease in California from 1995 to 2013. *Pediatr Surg Int*. 2018 Dec;34(12):1299-1303. doi: 10.1007/s00383-018-4363-9. Epub 2018 Oct 15. PMID: 30324568. [d] To our knowledge, there are no studies that report the population incidence of MRO of Hirschsprung disease.

© 2025 Rook JM et al. *JAMA Network Open*.

eTable 4. Candidate ICD-10 Codes for Meconium-Related Obstruction and Frequency of Co-Occurrence with Cystic Fibrosis and Hirschsprung Disease

		Concomitant Diagnoses, n (%)	
**ICD-10 Diagnosis Code (Definition)**	Cases, n	Cystic Fibrosis	Hirschsprung Disease
Codes Used to Identify MRO			
P76.0 (Meconium Plug Syndrome)	1,808	≤10 (<1.0%)	60 (3.3%)
E84.11 (Meconium ileus of Cystic Fibrosis)	29	29 (100.0%)	0 (0.0%)
E84.19 (Other Intestinal Manifestations of Cystic Fibrosis)	12	12 (100%)	0 (0.0%)
Codes Excluded as Identifiers of MRO			
P76.1 (Transitory Ileus of the Newborn)	1,534	≤10 (<1.0%)	≤10 (<1.0%) [a]
P76.2 (Intestinal Obstruction due to Inspissated Milk)	≤10	0 (0.0%)	0 (0.0%)
P76.8 (Other Specified Intestinal Obstruction of Newborn)	219	≤10 (<5.0%)	≤10 (<5.0%)
P76.9 (Intestinal Obstruction of Newborn, Unspecified)	647	≤10 (<2.0%)	21 (3.3%)
K56.0 (Paralytic Ileus)	17	0 (0.0%)	0 (0.0%)
K56.41 (Fecal Impaction)	≤10	0 (0.0%)	0 (0.0%)
K56.49 (Other Impaction of Intestine)	≤10	0 (0.0%)	0 (0.0%)
K56.5* (Intestinal Adhesions with Obstruction)	39	0 (0.0%)	0 (0.0%)
K56.6* (Other Unspecified Intestinal Obstruction)	64	0 (0.0%)	0 (0.0%)
K56.7 (Ileus, Unspecified)	141	0 (0.0%)	≤10 (<10.0%)

Excluded codes identified no additional cases of MRO of cystic fibrosis that were not already captured by the ICD-10 codes included for analysis. Excluded codes identified 27 potential cases of MRO of Hirschsprung disease that were not captured by the ICD-10 codes included for analysis. Given that these codes do not differentiate whether obstruction is due to inspissated meconium versus other mechanical obstruction, these cases were not considered MRO of Hirschsprung diseases. [a] Cells with 10 or fewer observations are reported as ≤10 per NIS guidelines. Abbreviations: MRO – meconium-related obstruction.

© 2025 Rook JM et al. *JAMA Network Open*.

eTable 5. Frequency of Meconium-Related Obstruction by Type Among Analytic Sample Versus Infants Excluded for Missing Data

**Meconium-Related Obstruction Type**	Included, n (%)	Excluded, n (%)	p-value
Total Patients	N=3,550,796	N=66,441	
MRO of Cystic Fibrosis	41 (0.001%)	≤10 (<0.02%) [a]	0.21
MRO of Hirschsprung Disease	60 (0.002%)	≤10 (<0.02%)	0.08
MRO of Prematurity	604 (0.02%)	≤10 (<0.02%)	0.33
MRO of the Term Infant	1,139 (0.03%)	33 (0.05%)	0.013

[a] Cells with 10 or fewer observations are reported as ≤10 per NIS guidelines. Abbreviations: MRO – meconium-related obstruction

© 2025 Rook JM et al. *JAMA Network Open*.

eTable 6. Incidence of Meconium Related Obstruction by Prematurity, Birth Weight, and Need for Surgery

	MRO of Cystic Fibrosis		MRO of Hirschsprung Disease		MRO Unspecified	
**Characteristics**	Cases per 100,000 [a]	p-value	Cases per 100,000 [a]	p-value	Cases per 100,000 [a]	p-value
**Total**	1.2		1.7		49.1	
**Prematurity**						
Extremely Preterm (<28w)	≤10 [b]	<0.001	≤10	<0.001	433.1	<0.001
Very Preterm (28w-31w6d)	≤10		≤10		438.1	
Moderately Preterm (32w-33w6d)	≤10		≤10		183.2	
Late Preterm (34w-36w6d)	≤10		5.2		132.5	
Term (≥37w)	0.8		1.4		35.3	
**Birthweight**						
Extremely low (<1,000 g)	≤10	<0.001	≤10	0.009	665.1	<0.001
Very low (1,000-1,499 g)	≤10		≤10		376.4	
Low (1,500-2,499 g)	≤10		≤10		121.9	
Normal (≥2,500 g)	1.0		1.5		39.1	
**Requiring Surgery**	0.5		0.5		1.8	
**Prematurity**						
Extremely Preterm (<28w)	0.0	<0.001	≤10	0.25	110.8	<0.001
Very Preterm (28w-31w6d)	≤10		≤10		66.7	
Moderately Preterm (32w-33w6d)	≤10		≤10		≤10	
Late Preterm (34w-36w6d)	≤10		≤10		≤10	
Term (≥37w)	≤10		0.5		≤10	
**Birthweight**						
Extremely low (<1,000 g)	≤10	<0.001	≤10	0.06	183.3	<0.001
Very low (1,000-1,499 g)	≤10		≤10		41.4	
Low (1,500-2,499 g)	≤10		≤10		≤10	
Normal (≥2,500 g)	0.4		0.5		0.5	

[a] Incidence reported as cases per 100,000 infants at-risk. For example, there are 433.1 cases of MRO unspecified per 100,000 infants born extremely preterm. [b] Cells calculated from 10 or fewer observations are reported as ≤10 per NIS guidelines. P-values calculated with chi-squared tests. Abbreviations: w – weeks; d – days; g – grams.

© 2025 Rook JM et al. *JAMA Network Open*.

eFigure. Incidence of Meconium-Related Obstruction Unspecified by Small for Gestational Age Status and Degree of Prematurity and Birth Weight

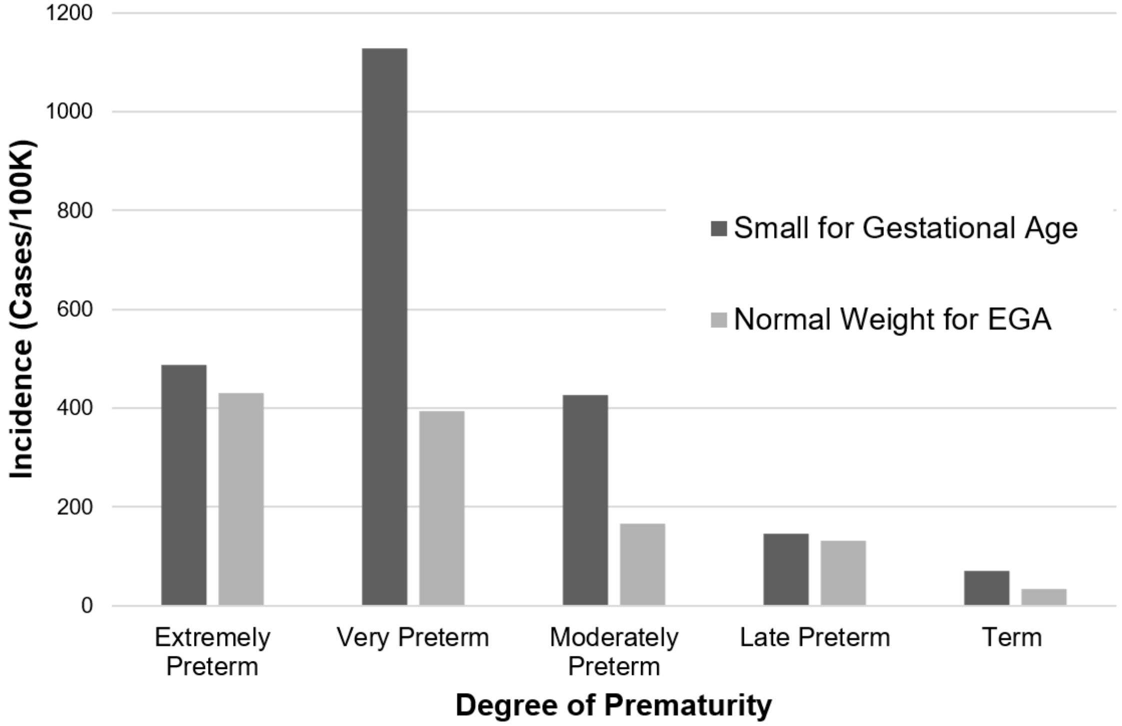

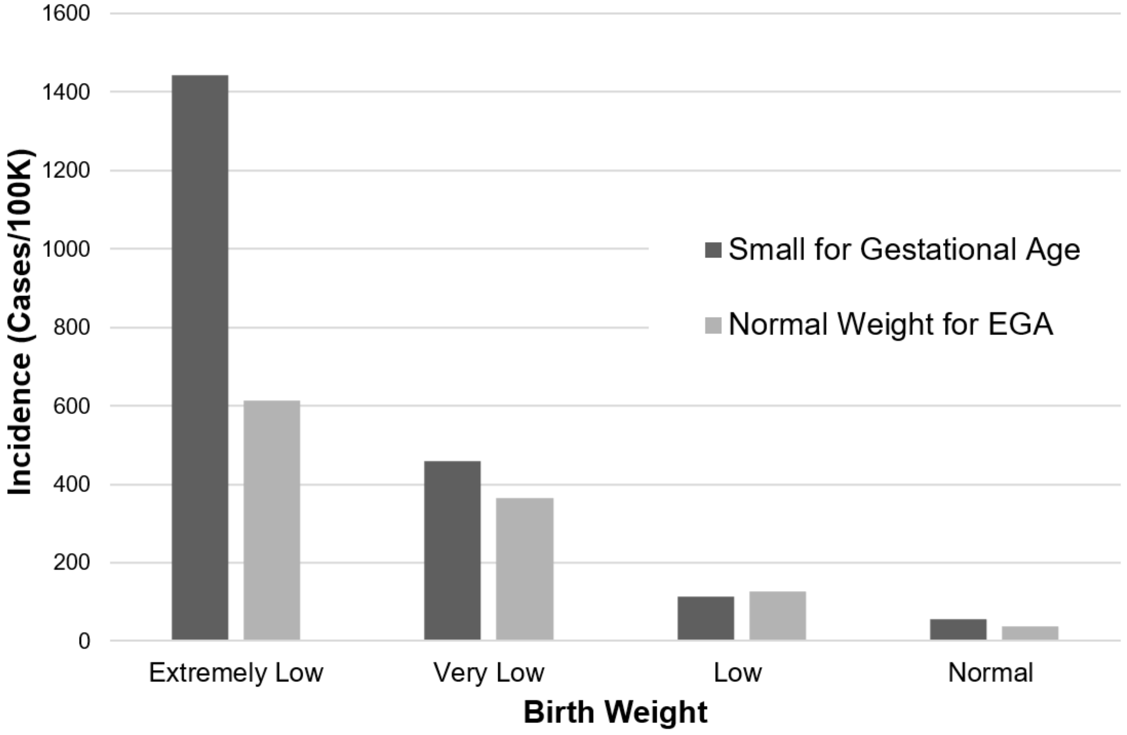

Definitions: Extremely preterm < 28 weeks; very preterm – 28 weeks to 31 weeks 6 days; moderately preterm – 32 weeks to 33 weeks 6 days; late preterm – 34 weeks to 36 weeks 6 days. Extremely low birth weight < 1,000 grams; very low birth weight – 1,000 to 1,499 grams; low birth weight – 1,500 to 2,499 grams; normal birth weight ≥ 2,500 grams. *Abbreviations*: EGA - estimated gestational age.

© 2025 Rook JM et al. *JAMA Network Open*.
