# Supplementary material for: Meconium-Related Obstruction and Clinical Outcomes in Term and Preterm Infants
Source: JAMA Netw Open. 2025 Feb 14;8(2):e2459557. doi: 10.1001/jamanetworkopen.2024.59557 (PMC11829230; doi:10.1001/jamanetworkopen.2024.59557)
Supplement: Supplement 2. — Data Sharing Statement [file jamanetwopen-e2459557-s002.pdf]

# Data Sharing Statement

Rook. Meconium-Related Obstruction and Clinical Outcomes in Term and Preterm Infants. *JAMA Netw Open*. Published February 14, 2025. doi:10.1001/jamanetworkopen.2024.59557

**Data**
**Data available:** No

**Additional Information**
**Explanation for why data not available:** Data described in this manuscript are available through HCUP. It cannot be shared without their consent.
